# Restless Genital Syndrome: A Case Report of the Challenging Diagnosis

**DOI:** 10.7759/cureus.61994

**Published:** 2024-06-09

**Authors:** Buse Cagla Ari, Elifnaz Sahin

**Affiliations:** 1 Neurology, Bahçeşehir University, Faculty of Medicine, Istanbul, TUR

**Keywords:** restless leg syndrome (rls), pramipexole, persistent genital arousal disorder, genital arousal, restless genital syndrome

## Abstract

Restless genital syndrome (RGS) is a rare disorder marked by paresthesia and discomfort in the genital area, akin to restless legs syndrome (RLS). While RLS typically affects the lower limbs, its impact on areas such as the bladder has been noted. RGS individuals exhibit sensory symptoms akin to RLS, including difficulty expressing sensations and a compulsion for genital rubbing. Thus, RGS is viewed as an atypical RLS presentation, characterized by genital sensory symptoms. Despite the rarity, this report details a successfully managed case using conventional RLS treatments. Numerous RGS aspects need clarification, including prevalence and treatment. Due to its distressing nature, sustained investigation is vital. Though lacking a standard treatment, our patient benefited from traditional RLS medication, hinting at shared mechanisms. Further research is vital for understanding and treating RGS effectively.

## Introduction

Restless genital syndrome (RGS) manifests as a clinical entity characterized by intrusive sensations within the genital region, marked by dysesthesia, paresthesia, and allodynia. Patients experiencing RGS may describe sensations similar to impending orgasm devoid of sexual desire or stimulation, while others report symptoms such as tingling, burning, or pain [1,2]. Despite its clinical significance, the frequency and underlying causes of RGS remain poorly understood, with existing data primarily derived from literature, predominantly comprising individual case reports. Moreover, RGS can affect individuals of the male gender as well [3]. Research elucidating its etiology and therapeutic approaches predominantly consists of case report publications [4]. Notably, RGS is conceptualized as a somatosensory dysfunction, implicating aberrations in sensory processing within the pudendal nerve's terminal branches and/or pelvic vasocongestion [5,6].

Accurately delineating the prevalence of RGS poses challenges, primarily due to potential social stigma and discomfort, particularly among women, in conservative societal contexts worldwide, when seeking medical evaluation for genital symptoms. Furthermore, variations in the interpretation of sexual desire among women further complicate efforts to ascertain RGS prevalence [3,6]. This study aims to contribute to the comprehension of this rarely observed and often underdiagnosed phenomenon by presenting a case of RGS, thereby promoting heightened awareness and understanding within the medical community.

## Case presentation

A 35-year-old female presented with a history of genital numbness and tingling that commenced approximately six months prior. The symptoms initially localized to the genital region before radiating sequentially to the right and, subsequently, to the left limb. These unpleasant sensations were accompanied by an uncomfortable pinprick feeling, necessitating movement of the lower extremities. Notably, the discomfort worsened prior to sleep onset, although without any reported issues during standing, movement, or sleep. The onset of symptoms consistently followed periods of rest, notably impeding her ability to initiate sleep at night. Notably, there was no alleviation of symptoms following orgasm. Despite consultations with neurosurgery, physical therapy, rehabilitation, and gynecology, clinical examinations yielded no significant findings. Her follicle-stimulating hormone (FSH), luteinizing hormone (LH), serum oestradiol, and progesterone levels were in normal ranges. Over time, symptoms initially confined to evenings began to manifest during daytime hours. The patient reported experiencing sexual arousal without corresponding voluntary sexual desire and spontaneous orgasms throughout the day, resulting in disruptions to daily functioning, difficulties in concentration, and feelings of anxiety and depression. Challenges in her professional life compounded her overall distress.

The patient's medical history included hypothyroidism, for which she was presently prescribed levothyroxine at a dosage of 125 mcg/day. There was no record of prior intra-abdominal surgery, and her familial medical history was unremarkable. The neurological examination revealed no abnormalities. Abdominal and urinary ultrasonography (USG), as well as lumbar and pelvic magnetic resonance imaging (MRI), showed no anomalies. Cranial MRI, conducted to exclude potential intracranial pathology, returned normal findings. Electrophysiological assessments, including needle electromyography (EMG) to evaluate peripheral nerve integrity, encompassing nerve conduction studies and motor unit action potential (MUAP) responses, demonstrated within-normal ranges in lower extremities. However, EMG for pelvic and perineal regions was omitted due to patient discomfort, precluding tolerance of the procedure. Blood tests disclosed a serum hemoglobin level of 12.3 g/dL, iron level of 112 µg/dL, and ferritin level of 125 ng/mL.

Following inconclusive investigations, pramipexole was initiated at a dosage of 0.125 mg once daily, considering the potential diagnosis of RGS. Subsequently, the dosage was gradually escalated to 0.250 mg three times daily, in response to partial amelioration of symptoms and sustained impairment in daytime functionality. Following adjustment of the dosage, symptomatic remission ensued, and the patient presently remains under continuous treatment surveillance.

## Discussion

This case report aims to heighten clinicians' awareness of RGS. When encountering a patient experiencing persistent sensations in the pelvic region without stimulation, clinicians should promptly consult a neurologist. Referring patients to urology, gynecology, or psychiatric clinics may lead to delays in diagnosis and discourage patients from seeking effective treatment. Importantly, RGS predominantly affects women; however, there is insufficient understanding regarding its onset, frequency, and gender distribution. Particularly in conservative regions, this lack of knowledge may cause considerable embarrassment and distress for patients. Notably, the literature reports a case wherein a patient suffered for 11 years before getting a diagnosis. Prolonging the time to diagnosis must be avoided at all costs to prevent further patient burden [3,4].

In 2001, Leiblum and Nathan classified numerous symptoms and labeled them as persistent sexual arousal syndrome (PSAS) [7]. Subsequently, recognizing the absence of a sexual context, PSAS underwent a renaming to persistent genital arousal disorder (PGAD) five years later [8]. A comprehensive study conducted on Dutch women with PGAD revealed a clinical association of the disorder with restless legs syndrome (RLS) and overactive bladder syndrome, indicating a dopaminergic mechanism [9]. Consequently, since 2009, the term RGS has supplanted PGAD. This semantic transition has the potential to engender confusion among clinicians, researchers, and patients, necessitating an elucidation of the taxonomic modification [2,8,9].

Facelle et al. subcategorized the etiology of PGAD into major and minor components. Major factors encompass psychological elements (associations with anxiety, depression, obsessive-compulsive disorder), biological factors (such as pudendal nerve neuralgia, Tarlov cysts, pelvic varices), and clinical clusters (including RLS and overactive bladder). Minor components include medication-induced causes, clitoral priapism, central nervous system pathologies (such as arteriovenous malformations, epilepsy, and stroke), dietary factors (like soy), and sleep-induced etiological factors [10].

In their systematic clinical study, Waldinger et al. unveiled significant prevalences detected in 18 Dutch women with PGAD: 67% of this cohort reported experiences with RLS, and one exhibited coexisting restless arms. Moreover, 67% manifested symptoms of overactive bladder, 55% presented with pelvis varices, and 39% experienced varices in the lower limbs. The study underscores these mutual clusters, highlighting the importance of not overlooking common pathogenesis. Furthermore, these clinically clustered syndromes may provide insights into establishing treatment guidelines [8-10].

The symptoms of RGS exhibit variability in character, duration, and location. Commonly reported symptoms include tingling, sensations of wetness, congestion, throbbing, and genital contractions. It has been observed that these symptoms can be triggered by either physical stimulation or psychological stress [10]. Trigger points may manifest unilaterally or bilaterally in areas including the vagina, clitoris, and above the pubic bone [7,9,10]. Diagnosis of PGAD relies on the criteria as follows: "feeling the physical sensations of sexual arousal without the complementary psychological component of desire that lasts for a prolonged period and does not resolve on its own", "feeling the onset of symptoms because of a sexual trigger", "having symptoms that do not resolve after one or even multiple orgasms", "experiencing arousal symptoms in the genitals for several hours or days without relief", "symptoms are seen as intrusive by the patient", and "experiencing at least moderate distress because of these unwanted symptoms" [8,10].

Although there is no established treatment protocol, our patient is prescribed a medication typically used for RLS due to the suspected underlying mechanism, resulting in beneficial effects. The efficacy of various treatments, including analgesics, benzodiazepines, dual selective serotonin and norepinephrine reuptake inhibitors, tricyclic antidepressants, dopamine agonists, anti-epileptic drugs, and/or transcutaneous electrical nerve stimulation (TENS), is observed and monitored based on the patients' circumstances [6,9]. Cognitive behavioral therapy currently stands as the most common treatment approach [4].

## Conclusions

In conclusion, RGS represents a relatively seldom-encountered disorder that can significantly impact individuals' well-being and functional capacity. A comprehensive clinical assessment is imperative for accurate diagnosis. It should be recognized as a phenotype of RLS, restless bladder, or restless abdomen and managed accordingly.
